# Localizing sensory processing sensitivity and its subdomains within its relevant trait space: a data-driven approach

**DOI:** 10.1038/s41598-021-99686-y

**Published:** 2021-10-13

**Authors:** Taraneh Attary, Ali Ghazizadeh

**Affiliations:** 1 Bio-Intelligence Unit, Electrical Engineering Department, Sharif Brain Center, Sharif University of Technology, Tehran, Iran; 2 School of Cognitive Sciences, Institute for Research in Fundamental Sciences, Tehran, Iran

**Keywords:** Psychology, Human behaviour, Machine learning

## Abstract

Sensitivity arising from enhanced processing of external and internal stimuli or sensory processing sensitivity (SPS) is known to be present in a sizable portion of the population. Yet a clear localization of SPS and its subdomains with respect to other relevant traits is currently lacking. Here, we used a data-driven approach including hierarchical clustering, t-distributed stochastic neighbor embedding (t-SNE) and graph learning to portrait SPS as measured by Highly Sensitive Person Scale (HSPS) in relation to the Big-Five Inventory (neuroticism, extraversion, openness, agreeableness, and conscientiousness) as well as to shyness, alexithymia, autism quotient, anxiety, and depression (11 total traits) using data from more than 800 participants. Analysis revealed SPS subdomains to be divided between two trait clusters with questions related to aesthetic sensitivity (AES) falling within a cluster of mainly positive traits and neighbored by openness while questions addressing ease of excitation (EOE) and low sensory threshold (LST) to be mostly contained within a cluster of negative traits and neighbored by neuroticism. A similar spread across clusters was seen for questions addressing autism consistent with it being a spectrum disorder, in contrast, alexithymia subdomains were closely fit within the negative cluster. Together, our results support the view of SPS as a distinct yet non-unitary trait and provide insights for further refinements of the current SPS concept and scales.

## Introduction

The way an individual reacts to stimuli depends on how those stimuli are mentally processed and internalized. About 20% of the population in the world are known to show enhanced sensitivity to environmental or emotional stimuli, a trait that has been recognized for more than two decades and distinguished from other personality and disorders as sensory processing sensitivity or SPS^1^. Almost from the beginning of its inception, SPS relationships with a number of traits and disorders such as introversion and neuroticism^1^, obsessive–compulsive disorder^2^, schizophrenia (SZ), post-traumatic stress disorder (PTSD)^3^, autism spectrum disorder (ASD)^3,4^, anxiety, depression, alexithymia^4,5^ as well as ‘the big-five’ personalities^6–8^ have been investigated. While SPS itself is not considered a disorder, it is considered to be a vulnerability factor^9^ with differential impacts on the well-being and success of an individual depending on the level of support^10,11^ or discouragement^5,12,13^ in the environment especially at childhood^9,14^.

Despite the long-term interest in SPS and its prevalence in the population, its relative placement with respect to other personalities and temperaments within the trait space and its coherence as a unitary construct^15,16^ are still debated. Indeed, recent studies have suggested at least three subdomains within SPS with differential relations to other traits^7^. These subdomains include EOE which addresses being mentally overwhelmed by external and internal demands, LST which refers to unpleasant arousal by sensory stimulation and AES which is related to awareness and sensitivity to aesthetic features. Among these, EOE and LST were found to be more closely related to neuroticism while AES was linked to openness^7^.

Here using the state of art in clustering algorithms, low dimensional embedding and graph learning, SPS is visualized in relation to other traits in a data-driven approach. In particular, data from a large and relatively diverse group of people was obtained using standard questionnaires addressing 10 traits besides SPS including the NEO-five factor inventory (NEO-FFI) consisting of neuroticism, extraversion, openness, agreeableness, and conscientiousness as well as other SPS relevant traits including shyness, alexithymia, autism quotient, anxiety, and depression. The 27-item HSPS questionnaire was used for measuring the SPS scale (Table 1). Results revealed two major clusters of traits to which SPS subdomains showed differential relations consistent with previous work^7^. In particular, EOE and LST were found to fall within a negative trait cluster which contained neuroticism, shyness, alexithymia, anxiety and depression while AES was found to belong to a positive trait cluster consisting of openness, extraversion, agreeableness and conscientiousness.

**Table 1:**
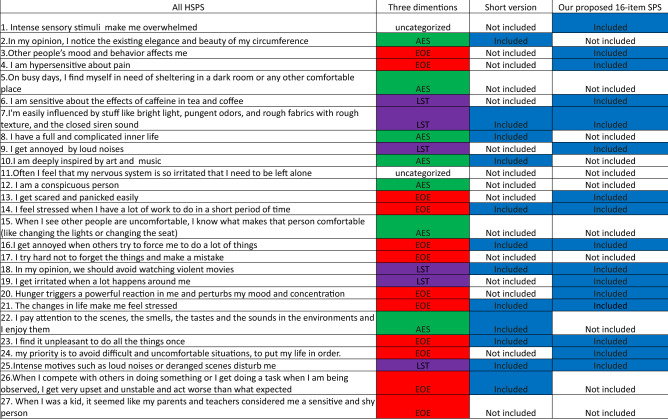
27-item HSPS questionnaire, its subdomains and its modified versions.

The original back-translated 27-questions HSPS (1st left column) along with the designation of the three subdomains AES, EOE and LST (25 questions) with 2 questions being uncategorized (2nd column). Short version of HSPS is shown in the 3rd column and our modified 16 questions version in the 4th column.

## Results

**Figure 1:**
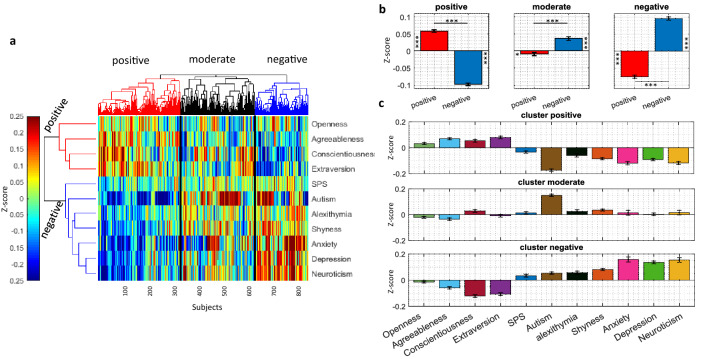
Trait z-scores across all subjects and hierarchical clustering of traits and subjects. (**a**) Clustergram of 837 subjects (columns) color-coded z-scores in 11 traits (rows) with rows and columns sorted by unsupervised HC (Methods). Subjects are clustered into three groups (positive, moderate and negative) and traits are clustered into two groups (positive and negative) as shown by dendrograms on top and on left, respectively. (**b**) Average z-score of positive traits and negative traits cluster in the three subject clusters of positive (t_327_ = 25.11), moderate (t_295_ = − 6.17), negative (t_212_ = − 21.81) for all *p* < 1e−13. *p < 0.05, **p < 1e−2, ***p < 1e−3 here and through-out (**c**) Average z-score of individual traits within each subject clusters. Error bars indicate s.e.m. here and through-out.

Figure 1a shows the scores of 837 subjects (Table S1) across the 11 traits color-coded by their z-score values (Methods). Each row represents the scores for one trait and each column is the score of one subject. Hierarchical clustering (HC) was used to sort both rows and columns based on the similarity of the traits across subjects and the similarity of subjects based on their trait scores, respectively. The HC grouped the traits into two distinct clusters. Cluster 1 traits included openness, extraversion, conscientiousness and agreeableness. Cluster 2 traits included autism, shyness, alexithymia, anxiety, depression, neuroticism and SPS. Roughly speaking, cluster 1 mostly consisted of positive traits (positive trait cluster) while cluster 2 consisted of negative traits (negative trait cluster). Conversely, subjects were also clustered into three groups at the global level. Group 1 had significantly higher positive trait scores (positive subject group), group 2 had moderate scores in both negative and positive traits (moderate subject group) and group 3 had significantly higher negative trait scores (negative subject group) (Fig. 1b). A closer look at the average z-scores showed that the scores for positive and negative traits to be significantly anticorrelated within each subject group (correlation in subject groups of positive, moderate, and negative were − 0.36, − 0.25, − 0.22, respectively with p < 2e−3). In the moderate subject group, the autism score seemed to be most prominent with relatively small scores in all other traits (Figs. 1c, S1). Interestingly, the absolute SPS score was not prominent in any of the three subject groups, suggesting that at the global scale SPS scores do not delineate a distinct subject group.

**Figure 2:**
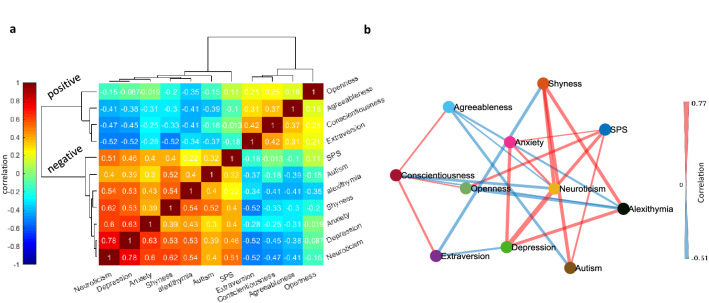
Pairwise correlation and graph representation of traits based on partial correlations. (**a**) Color-coded pairwise correlation matrix of 11 traits sorted by unsupervised HC. (**b**) The weighted undirected graph of 11 categories as extracted by the PC-stable algorithm. Color-coded edges surviving partial correlation (*p* < 0.01) are shown (red and blue indicating positive and negative correlations, respectively) and edge widths indicating the magnitude of partial correlation. Node locations are arranged by 2D embedding using t-SNE.

To investigate the trait relationships more formally, pairwise correlations between the 11 trait scores were examined. Figure 2a shows the pairwise correlation matrix, color-coded by correlation coefficient values with rows and columns sorted by HC. As can be seen, sorting of traits by HC nicely revealed the two positive and negative clusters as a block diagonalized correlation matrix.

The raw pairwise correlation between each two traits does not address whether the observed correlation between the two traits is indeed mediated by a third trait as a confounding factor. For example, the positive correlation between SPS and openness may be mediated by another node that has a positive correlation with both. To address this point, a graph learning algorithm was used to prune connections between nodes to only keep significant partial pairwise correlations that could not be explained by taking the contribution of all other nodes (^17^ PC-stable algorithm, Methods). Figure 2b shows significant partial correlations as edges between traits color-coded by the correlation sign and with edge widths representing the absolute magnitude of partial correlation. As expected the traits within a cluster tended to be connected with each other with positive weights and across clusters with negative weights. One of the few exceptions to this rule, was SPS which had positive connections to neuroticism, anxiety and autism in one cluster and positive connections to openness in the other cluster even when contributions of all other nodes were taken into account. A few other interesting observations include: (1) conscientiousness showed positive correlations to all other positive traits; openness, agreeableness and extraversion. The graph structure showed however that the pairwise correlations between the 3 aforementioned traits in Fig. 2a were arising because of their positive correlation with conscientiousness suggesting it to be a central node in the positive trait cluster. (2) For the negative traits, neuroticism had the largest number of positive correlations with the other members of the negative cluster and showed a strong negative correlation to conscientiousness. Formal analysis of graph centrality confirmed conscientiousness and neuroticism as two central nodes for positive and negative trait clusters, respectively (Table S2).

**Figure 3:**
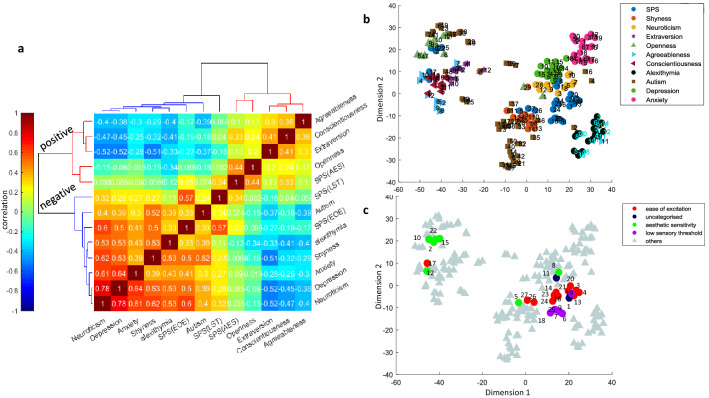
Localization of HSPS questions and its subdomains in the trait space. (**a**) Color-coded pairwise correlation matrix of 10 traits with the three subdomains of SPS sorted by unsupervised HC. (**b**) 2D embedding of all 210 questions across all 11 trait questionnaires using t-SNE. Questions belonging to each trait are shown with different symbols and colors. (**c**) Same as b but separately showing SPS subdomains AES, LST and EOE with other traits grayed out.

One reason for the fact that SPS has positive correlations to traits in both positive and negative clusters can be because it is not a unitary trait. Indeed, SPS is previously proposed to consist of three subdomains each addressed by a subset of questions in the 27-item HSPS questionnaire, namely ease of excitation (EOE), aesthetic sensitivity (AES) and low sensory threshold (LST) (2 questions remained uncategorized in the previous work^7^). It is possible that these different aspects of SPS have different relationships to negative and positive traits, thus explaining the borderline membership of SPS to negative and positive clusters. To address this point, first, we repeated pairwise correlations analysis with these three subdomains separated (Fig. 3a). We can see that AES has a strong association with openness to experience and joined the positive trait cluster using HC, however, EOE and LST had stronger correlations with the negative traits and stayed in the negative cluster. To further address the relationship of each component across all questionnaires, we used two-dimensional embedding of all 210 questions across the 11 traits using ^18^ t-SNE (Fig. 3b) with the pairwise correlation coefficients as the distance matrix between all questions and locations initialized by multidimensional scaling (Fig. S2, Methods). Results again confirmed the segregation of questions pertaining to negative and positive traits. Notably, for most of the traits, their questions tended to be well localized in the trait space, suggesting the distinctiveness of that trait relative to the rest. Furthermore, conscientiousness and neuroticism questions seemed to occupy pivotal positions within their parent clusters consistent with the previous conclusion about their graph centrality. Importantly for SPS, we see that some questions (6 questions) fall in the positive cluster while the rest (21 questions) fall in the negative cluster (Fig. 3b).

A closer look at the three SPS subdomains showed that most questions addressing AES fall in the positive trait cluster (5 out of 7 questions) while most questions addressing EOE (11 out of 12) and all questions addressing LST (6 out of 6) fall in the negative trait cluster (Fig. 3c). The two previously uncategorized HSPS questions were also located in the negative trait cluster. The examination of the HSPS questions in the positive cluster mainly belonging to the AES aspect showed that they fall very close to openness or conscientiousness. On the other hand, the questions in AES that could have negative connotations (questions 5 and 8, Table 1) were placed within the negative cluster. The HSPS questions in the negative trait cluster which mainly consisted of LST and EOE subdomains were bordered on three sides by neuroticism, shyness and alexithymia.

A similar spread for SPS in the trait space was observed using other variants of the original 27-item HSPS questionnaire^19,20^ (Fig. S3, Table 1). In all cases, HSPS questions addressing AES vs LST/EOE were divided between positive and negative clusters, respectively. AES was consistently found to be close to openness, while LST and EOE remained close to neuroticism. The relation to shyness and alexithymia showed some variation depending on the exact form of questionnaires in use.

In comparison, autism questions were also spread out in the trait space between positive and negative trait clusters regardless of the autism questionnaire version used^20^ or range of scoring (Figs. 3b, S3, S4). Autism is also often parsed into three subdomains. In this case, questions related to poor social skills were almost completely contained in the negative trait space (11 out of 12 questions) and were neighbored by shyness (Fig. S5a). Attention to details was close to the positive trait area with 6 out of 8 questions mostly neighboring openness. Poor communication was more spread out but was mostly contained in the negative trait space. This result is consistent with the conceptualization of autism as a spectrum of disorders rather than a unitary and well-defined trait.

In contrast, questions related to alexithymia, which is another trait with multiple subdomains seemed to be well concentrated and contained within the negative trait cluster neighbored by SPS and surrounding the central negative traits neuroticism and depression (Figs. 3b, S3, S4). Breakdown of alexithymia questions into its three subdomains, namely, difficulty in identifying feelings, difficulty in describing feelings and externally oriented thinking showed a nice pairwise separation with minimal intermingling between the subdomains. This suggests alexithymia to be a unitary trait with well-defined subcategories (Fig. S5b).

Our results indicate that HSPS questionnaire has components in both positive and negative trait space. The current formulation suggests SPS to be more like a trait spectrum similar to autism rather than a well-localized trait such as alexithymia. The low dimensional embedding of traits (Fig. 3b) may be used for modification of HSPS questionnaire for better localization within the trait space. For instance, removing HSPS questions that were not linearly separable from other traits. In particular, the positive part of AES (questions 2, 10, 15 and 22) was not linearly separable from openness (SVM classification error: 25%), questions 17 and 12 were not separable from consciousness (SVM classification error: 21.61%), questions 11 and 8 were mixed with neuroticism (SVM classification error: 7.67%), and questions 5, 26 and 27 were too close to shyness (SVM classification error: 26.67%). Removing these questions resulted in a modified SPS questionnaire with 16 questions with improved alpha Cronbach of 0.92 compared to the 0.82 of the original 27 HSPS questionnaire (Tables 1, S3). Furthermore, the two-dimensional embedding of SPS subdomains (Fig. 3c) revealed that the uncategorized question 1 and question 19 from LST were well within EOE so one may switch their membership.

**Figure 4:**
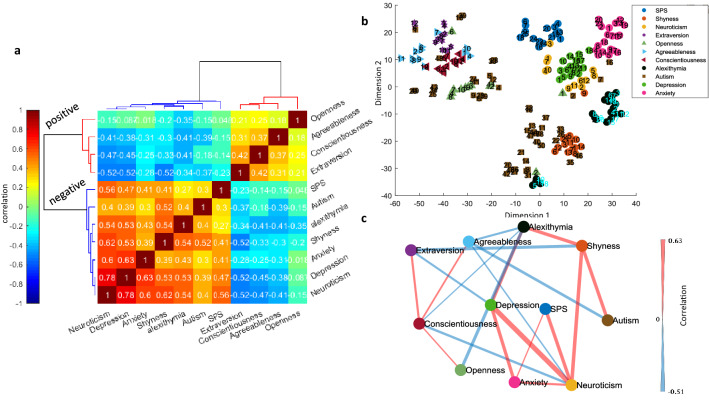
Modified 16-question version of HSPS shows better localization in the trait space. (**a**, **b**) same format as Figs. 2a and 3b, respectively but for the modified version of the HSPS questionnaire with AES component mostly removed. (**c**) same format as Fig. 2b but for the SPS score based on the modified HSPS questionnaire.

The correlation matrix using this modified HSPS questionnaire and sorted by HC still showed the two ‘positive’ and ‘negative’ clusters with the SPS in the negative cluster (Fig. 4a). However, the positive correlation with openness was greatly reduced (0.048 vs 0.11). Low dimensional embedding of the trait space now showed all questions of SPS to be within the negative cluster and still neighbored by neuroticism (Fig. 4b). Note that the modified SPS questionnaire also increased the separation of SPS from autism, shyness and alexithymia. Graph analysis confirmed modified SPS not to be connected with openness but to retain a strong positive partial correlation to neuroticism (Fig. 4c). Neuroticism and conscientiousness kept their central position based on their connectivity in negative and positive clusters, respectively. Note that while our modified HSPS questionnaires enhances the relationship between SPS and neuroticism nevertheless it locates SPS as a distinct trait which is well separated from neuroticism in the trait space (SVM classification error between SPS and neuroticism was 4% compared to 7.67% in the original version, Fig. 3b). Use of this modified HSPS questionnaire still resulted in three subject groups with positive, negative and moderate scores (Fig. S6) but with some group switching among subjects most notably with subjects migrating out from the moderate group into positive or negative groups (Table S4).

**Figure 5:**
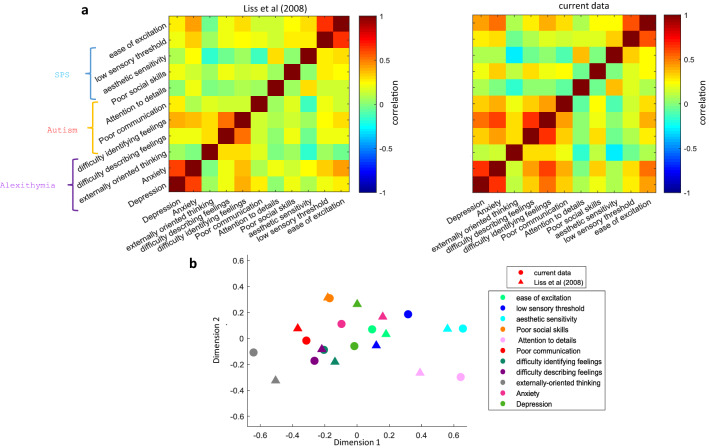
Trait score comparison with previously published data. (**a**) Color-coded correlation matrix of anxiety, depression, and subdomains of HSPS, autism, and alexithymia for Liss et al.^5^ and current data. (**b**) 2D embedding of trait scores using MDS (Methods) using the correlation matrix provided by Liss et al. (triangles) and the current data (circles) with colors showing different traits or their subdomains.

To ensure that our trait scores and localizations are comparable with previous reports and are not adversely affected by the subjects' ethnicities (Iranians) or translated questionnaires (Persian translation), we compared our results with previously published data. In particular, we used the correlation matrix reported by Liss et al.^4^ who also used a set of questionnaires including HSPS, autism, alexithymia, anxiety and depression. Figure 5a shows the comparison of our correlation matrix with Liss et al. across the traits and subcategories common to both studies. As can be seen, there is a qualitative agreement between both studies in terms of pairwise correlations found between traits. In particular, there are prominent positive correlations between anxiety and depression, EOE and LST of HSPS and poor communication and difficulty in identifying feelings in both datasets. The negative correlation between AES with externally-oriented thinking and poor communication is also seen in both datasets. Importantly, the 2D embedding and visualization of both correlation matrixes resulted in similar localization of traits, even down to the examined subdomains of SPS and alexithymia (Fig. 5b).

**Figure 6:**
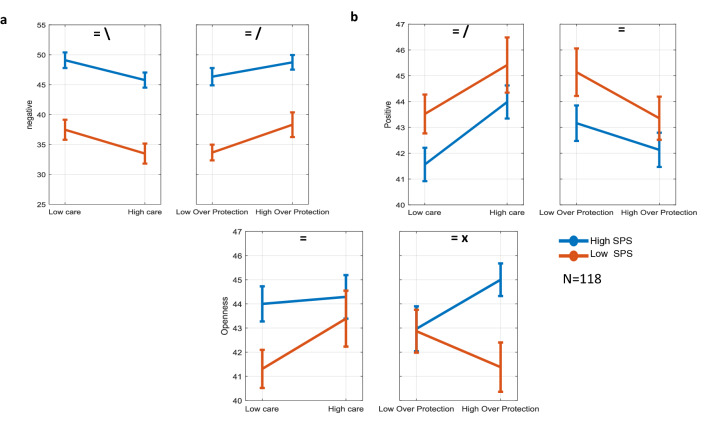
Effect of SPS and PBI (care and overprotection) on average scores of negative and positive clusters and on openness scores. (**a**) Negative trait score: main effect of SPS F_1,114_ > 58.87, *p* < 0.0, main effect of care F_1,114_ = 5.95, *p* = 0.01, SPS x care interaction F_1,114_ = 0.06, *p* = 0.80, main effect of overprotection F_1,114_ = 5.56, *p* = 0.02, SPS x overprotection interaction F_1,114_ = 0.59, *p* = 0.44, (**b**) positive trait score: main effect of SPS F_1,114_ > 4.01, *p* < 0.04, main effect of care F_1,114_ = 7.96, *p* = 0.005, SPS x care interaction F_1,114_ = 0.09, *p* = 0.76, main effect of overprotection F_1,114_ = 3.36, *p* = 0.06, SPS x overprotection interaction F_1,114_ = 0.27 , *p* = 0.60, (**c**) openness score using the original 27-item HSPS: main effect of SPS F_1,114_ > 0.74, *p* < 0.05, main effect of care F_1,114_ = 1.7, *p* = 0.19, SPS x care interaction F_1,114_ = 0.97, *p* = 0.32, main effect of overprotection F_1,114_ = 0.09, *p* = 0.76, SPS x overprotection interaction F_1,114_ = 3.9, *p* = 0.05. = , significant main effect of SPS, \ significant main effect of parental care or overprotection, x significant interaction.

It is believed that the negativity score for SPS individuals is significantly modulated by their childhood environments and in particular their parental experience^1,12^. To investigate this issue and further validate our results in comparison to previous findings, we obtained parental bonding scores (using Parental Bonding Instrument questionnaire or PBI) from a subset of our original participants (Methods). Consistent with previous findings^5^, we found strong main effects of SPS and both PBI scores on depression and anxiety but minimal crossover interactions (Fig. S7a,b). The correlation coefficient matrix between SPS, depression, anxiety and parental care (Table S5) was consistent with the values reported previously^5^. Additional analysis using the average of negative and positive trait scores, based on the trait clusters revealed previously, also replicated the significant main effects of SPS and both PBI scores and the lack of interactions (Fig. 6a,b). This was also true for neuroticism as the central node in the negative cluster and one of the most related traits with SPS (Fig. S7c). Overall individuals with high SPS scores scored higher on negative and lower on positive trait scores compared to individuals with low SPS scores. Better parental care increased positive and decreased negative trait scores for all subjects while higher parental overprotection had the opposite effects. However, there was one exception in crossover interaction of SPS and PBI which was observed with regards to the openness scores. Results showed a significant interaction between SPS and overprotection on openness scores. Notably, while overprotection decreased openness for individuals with low SPS scores consistent with its effect on the average score for positive traits, it had the opposite effect and improved openness scores for highly sensitive persons. Thus individuals with high SPS scores tended to have higher openness at higher overprotection levels (Fig. 6c). For the other traits in the positive cluster, the effects of SPS and PBI were consistent with the average scores (Fig. S8).

## Discussion

Despite long-term interest in SPS as a distinct and prevalent personality trait, its relation to other closely related traits and its level of coherence as a unitary trait remains controversial. Here, we examined the relationship of SPS to 10 other traits which were previously considered to be relevant for SPS. These traits included neuroticism, extraversion, openness, agreeableness, and conscientiousness (the ‘big-five’ personality dimensions) as well as shyness, alexithymia, autism quotient, anxiety, and depression. While individuals with high SPS scores formed a sizable chunk of our surveyed individuals (41.46%)^6^, SPS scores did not demarcate a distinct group within the population nor were prominent in any of the three globally detected subject groups (Fig. 1). In contrast other traits such as neuroticism, consciousness and autism scores were differentially present in the three subject groups demarcated by hierarchical clustering (Fig. 1b,c). Importantly, using low dimensional embedding and visualization of the trait space, we found that SPS subdomains AES and LST/EOE were divided between positive and negative trait clusters, respectively. The positive cluster contained openness, extraversion, conscientiousness and agreeableness. The negative cluster contained autism, shyness, alexithymia, anxiety, depression and neuroticism. However, while AES was mostly intermixed with openness and hard to distinguish from it, LST and EOE occupied distinct positions in the negative cluster bordered most consistently by neuroticism (Figs. 3, S3). Graph learning on the 11 examined traits revealed a central position for neuroticism and conscientiousness in the negative and positive clusters, respectively (Table S2). Furthermore, SPS had a strong and significant positive correlation with neuroticism even when the effects of other traits were taken into account using partial correlations (Fig. 2b).

Using low dimensional embedding and visualization techniques, our results confirmed many of the past intuitions and findings about the relation of SPS to other traits. From its inception,^1^ considered emotionality (neuroticism) as a likely confounder of the newly conceptualized SPS trait. They however showed that despite significant correlations SPS seemed distinct from neuroticism. Indeed, visualization of the trait space showed SPS to be close to neuroticism yet distinct from it (SVM classification error < 8%). This closeness was mostly observed for EOE and LST subdomain of SPS consistent with the previous reports^4,7^. Interestingly, we also found a close relationship between EOE and LST subdomains with shyness and with alexithymia consistent with previous reports. It is indeed possible that high irritability and being overwhelmed by external stimuli leads to social anxiety and withdrawal (i.e. shyness)^12^. The overwhelming experiences may also cause an inability to correctly identify and describe the resulting emotions resembling alexithymia like symptoms.

The 2D visualization may provide insights for further refinement of current HSPS questionnaires depending on particular objectives. We have proposed one such modification with the objective of making the HSPS being better localized within the trait space similar to alexithymia (another multi-domain trait). Given the fact that (1) EOE and LST subdomains were largely contained in the negative cluster and separate from the AES and (2) AES was not found to be very distinct from openness (SVM classification error: 25%), we created a modified HSPS questionnaire which had the AES questions mostly excluded (Table 1, S3). Trait clustering and low dimensional embedding using this modified HSPS questionnaire again revealed the same two major positive and negative clusters (Fig. 4a). While using the modified HSPS questionnaire, SPS was still found to be a borderline trait (Fig. 4a,b), nevertheless, this time SPS was well contained within the negative trait (Fig. 4b) and did not show a significant positive correlation with openness (Fig. 4c). In the negative cluster, SPS was again bordered with neuroticism which was the central trait in the negative cluster (Fig. 4b). The modified HSPS also showed a higher alpha Cronbach compared to the original scale (0.92 vs 0.82). Further work by psychologists in the area is required to validate and prune the HSPS questionnaire beyond the data-driven approaches taken in this study.

It is indeed the case for many personality traits to have both positive and negative manifestations in behavior. This is also true for SPS which endows a person with higher sensitivity to subtleties both for aesthetic dimensions as well as the harrowing aspects of life. Given the original emphasis on the positive aspects of SPS which was mainly due to the AES subdomain, our modified HSPS may miss important aspects of SPS related to openness and creativity^21^. Indeed, we also see a generally higher openness in individuals with higher SPS scores compared to others (Fig. 6c) mostly due to the inclusion of AES subdomain. Individuals with high openness tend to have a strong aesthetic sense^22^ and high openness may be related to the function of the central dopaminergic (DA) system, which is implicated in rewards sensitivity^7,23^. Indeed, research into the neural substrates of stimuli discrimination showed a higher activation in visual attentional areas (right claustrum, left occipitotemporal, bilateral temporal and medial and posterior parietal regions) for subtle decisions in high SPS individuals^24^. Other research indicated that SPS individuals can show higher activations in brain regions involved in awareness and empathy (insula, inferior frontal gyrus), memory, and self-other processing (i.e., premotor area, cingulate, medial and dorsolateral prefrontal cortex)^24–27^ and enhanced functional connectivity among attentional and limbic network in resting state^28^. These results are consistent with highly sensitive persons being more aware and more intensely affected by others’ moods^9^ and having an enhanced depth of processing^14^. On the negative side, these could explain why highly sensitive people score higher on anxiety, depression and neuroticism compare to people with low SPS scores (Fig. S7).

From the inception of the SPS concept, it was shown that SPS individuals with low quality of childhood have disproportionately higher negative affectivity and shyness compared with low SPS individuals with similar childhood quality (crossover interaction)^1^. Later research on SPS children showed that parenting quality plays an important role in behavioral problems during adulthood with long-lasting impacts^9,10,25^. Nevertheless, studies that have found generally higher anxiety and depression levels for high SPS individuals only found marginal interactions with two measures of parental environment including the parental care and overprotection^5^. Consistent with these, our results also showed minimal interactions between PBI dimensions and SPS on depression, anxiety, neuroticism (Fig. S7) and the average scores of  negative and positive traits (Fig. 6). However, we found a significant crossover interaction between SPS and PBI on openness (Fig. 6c). In particular, we found that overprotection which has normally adverse effects on trait scores (increasing negative and decreasing positive scores) tends to increase openness scores for highly sensitive individuals. Such interactions may be related to the significant neural correlations across temporal/parietal as well as limbic/memory areas to emotional vs neutral stimuli as a function of SPS and childhood quality interactions^26^.

In summary, our results revealed the relative position of SPS with respect to two main trait clusters formed by positive and negative traits and confirmed the distinctiveness of the negative subdomains of SPS from neuroticism. We note that our findings are of a correlational nature and proximity of traits to each other does not by itself imply a causal relation between them. The question of whether the proximity of SPS in the trait space causes a higher predisposition for the neighboring traits such as neuroticism and depression, requires longitudinal studies along with interventions to elucidate the direction of causal relations.

## Materials and methods

### Subjects

Request for participation was advertised across multiple university campuses as well as on social media such as Telegram. A total of 875 people participated in filling our questionnaires voluntarily using an online google form. Subjects were only required to provide a valid email to be able to take part in the study. Providing any personally identifiable information (including name, comment about diseases, or place of work) was optional. The subjects’ data were collected and stored in an encrypted hard drive and data analysis was blind to personally identifiable information. After submitting the form, subjects received their standard scores for all the psychology questionnaires via email along with a disclaimer to refer to specialists for expert advice in case of any concern. According to the self-reports in the online form, 83 subjects had various physiological or psychological disorders. Among those, 45 subjects reported physiological disease or disorders (e.g. skin diseases, high cholesterol, asthma, diabetes, digestive system disease, Lumbar disc, Sinusitis) while 38 subjects reported mental or brain related problems including obsessive-compulsive disorder (OCD), Bipolar disorder, Epilepsy, attention-deficit disorder (ADD), head surgery, multiple sclerosis (MS), pituitary tumor, Sjogren syndrome, multiple endocrine neoplasia (MEN) syndrome, Devic's disease. The group with mental or brain related problems was excluded from the main analysis. Thus, the final sample consisted of 837 subjects in total (68.57% women) with a mean age of 28.12 (SD = 9.64), (Iranian: 66.06%, Afghan: 2.51%, Arab: 0.25%, Kurdish: 6.33% Turkish: 15.53% others: 9.31%, Table S1). Data was collected from September 2019 until January 2020. To address the effect of parenting quality (parental care and parental overprotection) on results, later we asked participants to fill the PBI questionnaire. 118 of our original subjects (72% female) with a mean age of 31.3 (SD = 9.38) filled the questionnaire with about half (45.68%) filling out the mother form first. 53% had high scores on care and 50% of individuals had high scores on over protection of father. 37.9% had high scores on care and 42% of individuals had high scores on over protection of mother. We used the average of both mother and father forms for our analysis and investigated the interaction between SPS and parental quality on two clusters of positive and negative traits.

All study procedures were approved by Institute for Research in Fundamental Sciences (IPM) ethics committee (permit# 99/60/1/6115). Subjects gave online informed consent after reading the study information on the first page by providing their email address and clicking to start the questionnaires. All study procedures were in accordance with the Declaration of Helsinki.

### Questionnaires

We addressed 11 behavioral traits as well as parental bonding using eight questionnaires: Highly Sensitive Person Scale (HSPS), NEO Five-Factor Inventory (neuroticism, extraversion, openness, agreeableness, and conscientiousness), Revised Cheek–Buss Shyness Scale (RCBS), Toronto Alexithymia Scale (TAS-20), Adult Autism Spectrum Quotient (Adult AQ), Beck Anxiety Inventory (BAI), The Parental Bonding Instrument (PBI) and Goldberg’s Depression scale. Persian translation of questionnaires with previously established reliability and validity were used for all the 12 traits examined in this study. This included the ‘Big-five’ (NEO-PIR)^29^ ,shyness (Revised Shyness Scale)^30^, Depression (Goldberg's Depression)^31^, autism quotient (Adult AQ)^32^, anxiety (BAI)^33^, alexithymia (TAS-20)^34^, parental quality (The Parental Bonding Instrument)^35^ and SPS (HSPS)^36^ with the addition of two uncategorized questions which will be discussed below.

The Highly Sensitive Person Scale (HSPS) is used to distinguish people with sensory processing sensitivity and is a self-report questionnaire with 27 questions to assess high sensory processing^1^. A person with sensory processing sensitivity is proposed to be “particularly sensitive to subtle stimuli, easily overstimulated, prone to ‘pause to check’ in a novel situation, and prefer to reflect and revise their cognitive maps after an experience”^12^. HSPS questionnaire scale ranges from 1 to 7, lowest to highest respectively. The HSPS questioner with 25 questions (without the uncategorized questions in the 27-item questionnaire shown in Table 1 column 2) was previously translated to Persian and validated^36^. We added the Persian translations of the two uncategorized questions to use the full 27-item scale. For this purpose, standard translation/back-translation procedure by professional translators was followed. At each stage, two psychologists reviewed and edited the questions for face and content validity and the back-translated SPS questions were cross-checked with the original one. Split-half reliability of our final 27-item Persian questionnaire was 0.74. The Alpha Cronbach of the translated Persian HSPS questionnaire used in this study was 0.82 which is comparable to the original English version (0.87)^1^ as well as other international translations such as Russian alpha = 0.83, Spanish alpha = 0.85 or Afrikaans alpha > 0.8)^1,37–40^. Also, in this study, the Alpha Cronbach of the three-dimension^7^ version of HSPS was 0.80 and the short version^19^ of HSPS was 0.66. 41.46% of our participants were found to score high in SPS using a previously introduced threshold^6^ with the 27 item-HSPS questionnaire and after excluding people with high scores in depression (score > 53)^31^ as previously suggested^5,12^.

The Toronto Alexithymia Scale (TAS-20) with 20 questions was used to asses a person's ability to express emotions. A person with Alexithymia is proposed to “have reduced ability for describing emotions, emotion recognition and thinking with an external orientation”^41^. TAS-20 scale ranged from 1 to 5, strongly disagree to strongly agree, respectively. The Alpha Cronbach of the translated TAS-20 questionnaire used in this study was 0.85.

The Autism Spectrum Quotient (AQ) questionnaire with 50 questions assesses the severity of autistic traits symptoms by measuring the person’s ability in communication and social skills. “Definitely agree” or “slightly agree” responses scored one point, “Definitely disagree” or “slightly disagree” responses scored zero point^42^. The Alpha Cronbach of the translated AQ Autism questionnaire used in this study was 0.60. Also, the Alpha Cronbach of autism questionnaire with three components^43,44^ and the short version^20^ in this study was 0.61, and 0.41, respectively. We note that we changed the autism scores for our main analysis from 0 and 1 to 1, 2, 3 and 4 in order to have score ranges more similar to other questionnaires. However, using the original scores resulted in qualitatively similar trait localization (Fig. S4).

The NEO-FFI is a personality questionnaire^45^ assessing 5 personality traits of ‘neuroticism’ (When I'm under a great deal of stress, sometimes I feel like I'm going to pieces.), ‘extraversion/introversion’ (I am a cheerful, high-spirited person), ‘openness to experience’ (I often try new and foreign foods), ‘agreeableness’ (If necessary, I am willing to manipulate people [reverse question]), and ‘conscientiousness’ (I try to perform all the tasks assigned to me conscientiously.). The test consists of 60 questions with a scale ranging from 1 to 5 corresponding with strongly disagree to strongly agree, respectively for all 5 personalities tested. The alpha Cronbach of translated NEO-FFI questionnaire were 0.87, 0.78, 0.60, 0.69 and 0.83 for neuroticism, extraversion, openness, agreeableness, and conscientiousness, respectively in this study.

Revised Cheek–Buss Shyness Scale (RCBS) is a 14 item questionnaire to assess shyness which is described as having low self-stem, lack of determination, social distress and avoidance. This questionnaire scale ranged from 1 to 5 strongly disagree to strongly agree, respectively^46^. The Alpha Cronbach of translated RCBS questionnaire was 0.87 in this study.

Goldberg’s Depression Scale^47^ is a self-report measure with 18-item self-rating scale. The response scale ranges from 0 to 5, from not at all to very much, respectively. The Alpha Cronbach of translated depression questionnaire was 0.94 in this study.

The Beck Anxiety Inventory (BAI) is a 21-item questionnaire assessing common symptoms of anxiety such as difficulty in breathing, losing control and unable to relax^48^. The response scale ranges from 0 to 3, from not at all to severely-it bothered me a lot. The Alpha Cronbach of translated BAI questionnaire was 0.91 in this study.

The Parental Bonding Instrument is a self-report measure with 25 item questionnaire about perception of individual’s parents during the first 16 years of their life^49^. It is used to assess the parental quality and has two dimensions of care and over-protection. It has one form for mother and one for father. The alpha Cronbachs of translated PBI in this study were 0.89, 0.94 for care of mother and father, respectively; and was 0.91 for over protection for both parents.

### Data analysis

All data analyses were done by MATLAB 2017b. For our analysis, the scores for each question were z-scored across subjects. Initial grouping of 837 subjects and 11 traits were done using hierarchical clustering (MATLAB Clustergram, weighted Linkage Value, and distance correlation as row and column PDist, and without optimal leaf order). Low dimensional embedding was done using Nonclassical Multidimensional scaling (MDS, Fig. S2). The results of MDS were used as initialization for t-distributed stochastic neighbor embedding (t-SNE, 80 exaggeration: 80, learning rate: 100, MaxIter: 5000, perplexity: 10). The distance used in hierarchical clustering and for low dimensional embedding was Pearson’s correlation. For the classification of t-SNE data, a support vector machine (SVM) classifier was used. Percentage of classification error was obtained and reported. Classification error was defined as number of samples incorrectly classified divided by the total number of samples.

The PC-stable algorithm was used for graph learning with traits as nodes (e.g. Figure 2b). This algorithm aims to extract the causal graph skeleton based on conditional independence tests (using partial correlations). Blue and red edges show negative and positive correlations, respectively. The width of each edge was set to the lowest significant partial correlation between node pairs representing the net pairwise correlation between two nodes. The PC algorithm was implemented using code provided from^50^ and by considering *p*-value < 0.01 for the significance of an edge. Node centrality in the graph was quantified using MATLAB Centrality (c1, ‘degree’, ‘Importance’, c1.Edges.Weight) and reported in Table S2.

### Statistical power and tests

The sample size for having 80% power in detecting 0.1 change in correlation coefficient at *p*-value <0.05 using the fisher-z transform was calculated to be 783^51^ (http://sample-size.net/correlation-sample-size/). Our final sample size after exclusion of participants with mental disorders was 837 which is comfortably beyond this minimum sample size. Two-way anova was done using MATLAB ANOVAN and post hoc test (Tuckey’s hsd) was used to examine main effects and interactions (Figs S7, S8, 6). T-tests were used for individual comparisons (e.g. Fig. 1b). Unless otherwise noted *p* values < 0.05 was considered significant.

### Alpha Cronbach calculation

For Alpha Cronbach calculation, we used code from (https://ch.mathworks.com/matlabcentral/fileexchange/7829-cronbach-s-alpha) with this formula of coefficient alpha: $\rho_{T} = \frac{k}{k - 1}\left( {1 - \frac{{\sum\nolimits_{i = 1}^{k} {\sigma_{i}^{2} } }}{{\sigma_{x}^{2} }}} \right)$, where k is the number of questions, I is the number of subjects, $\sum\nolimits_{i = 1}^{k} {\sigma_{i}^{2} }$ is a sum of the items variance, and $\sigma_{x}^{2}$ is a variance of the items.

## Data and code availability

All data needed to evaluate the conclusions in the paper are present in the paper and/or the Supplementary Materials. Data and code can be made available at reasonable request.

## Supplementary Information

Supplementary Information.
