# Supplementary material for: Localizing sensory processing sensitivity and its subdomains within its relevant trait space: a data-driven approach
Source: Sci Rep. 2021 Oct 13;11:20343. doi: 10.1038/s41598-021-99686-y (PMC8514528; doi:10.1038/s41598-021-99686-y)
Supplement: Supplementary file 1 — Supplementary Information. [file 41598_2021_99686_MOESM1_ESM.pdf]

Supplemental Figures

Localizing sensory processing sensitivity and its subdomains within its relevant trait space: a data driven approach

Taraneh Attary[1], Ali Ghazizadeh[1, 2*]

[1]Bio-Intelligence Unit, Sharif Brain Center, Electrical Engineering Department, Sharif University of Technology, Tehran, Iran

[2]School of Cognitive Sciences, Institute for research in fundamental sciences, Tehran, Iran

Sup Figure.1

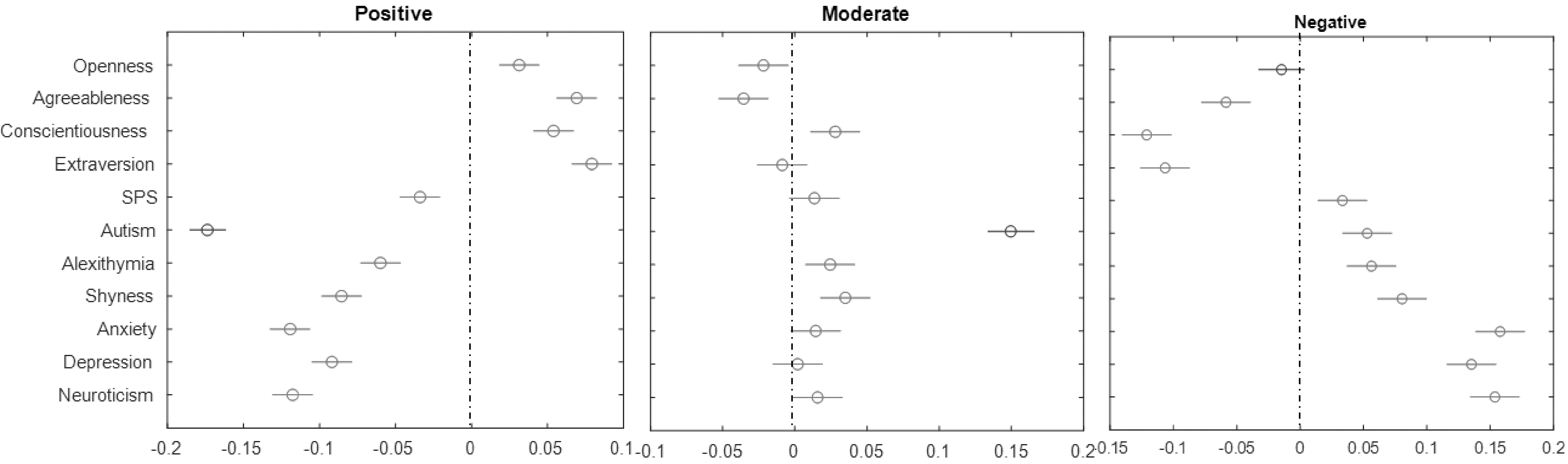

Supplementary Figure 1: Pairwise contrasts for the individual trait scores within the three subject groups. Non-overlapping error bars across any two traits indicate significant difference for all pairwise comparisons with correction for multiple comparisons using Tukey's method (hsd, $p<0.05$). For the positive group, positive trait scores were significantly positive and negative trait scores were significantly negative and vice versa for the negative group. Note that in the moderate group the scores of all traits but autism were small.

Sup Figure.2

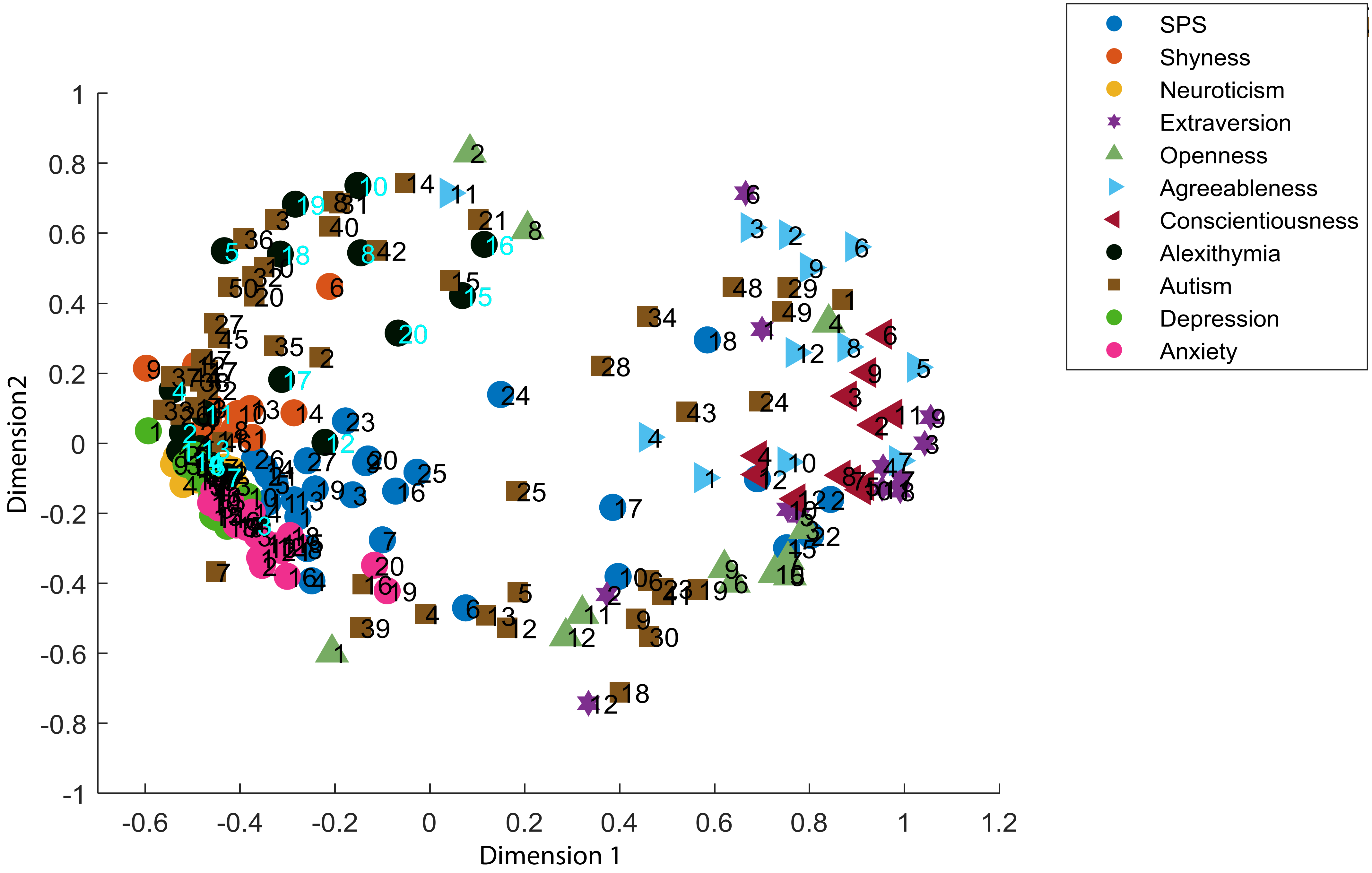

Supplementary Figure 2: Initialization of 2D embedding locations of traits using MDS. 2D embedding of all 210 questions across all 11 trait questionnaires using MDS. Questions belonging to each trait are shown with different symbols and colors. These locations were used as the best initial guess for the initialization of t-SNE (Figure 3b).

Sup Figure.3

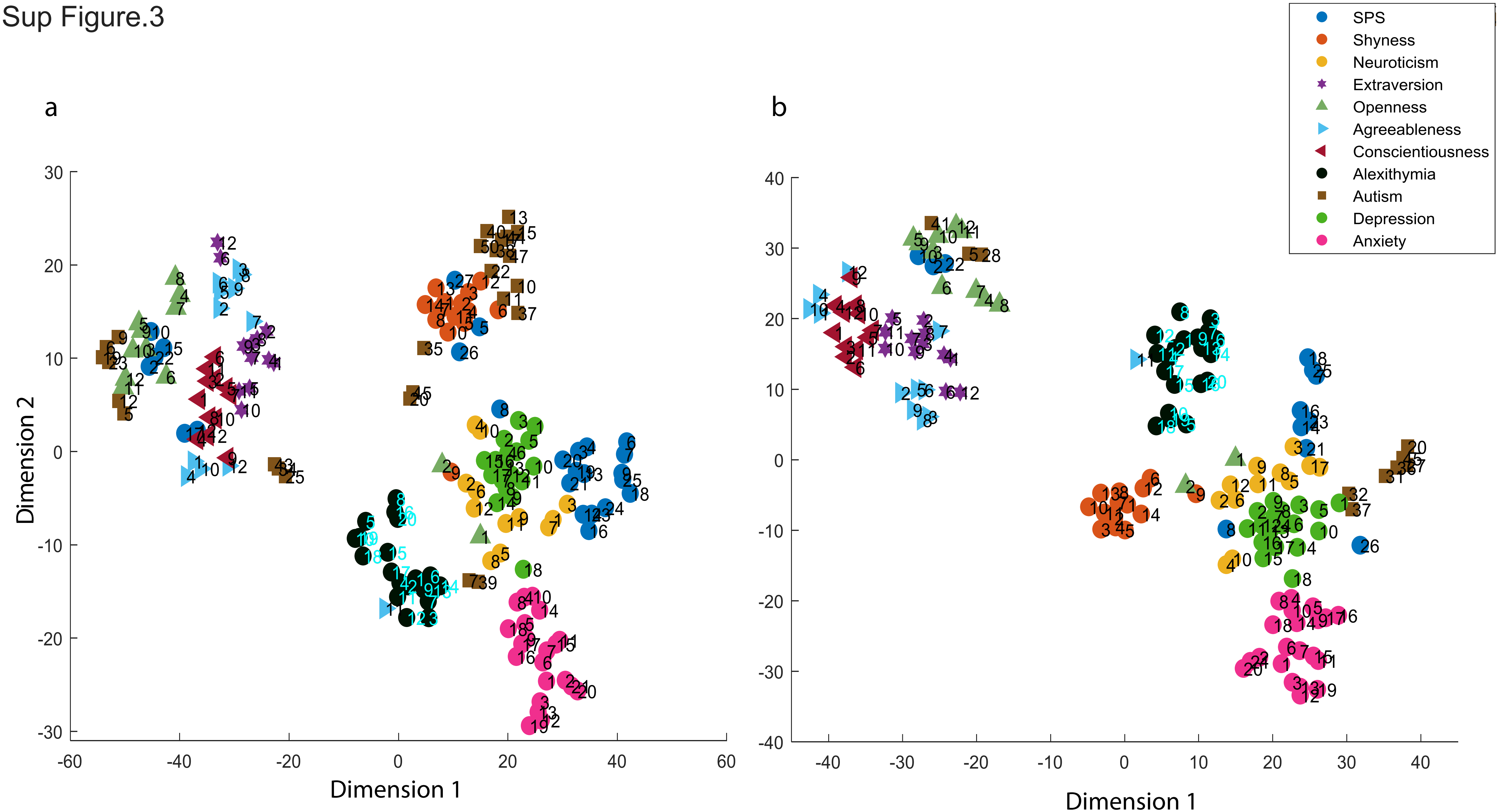

Supplementary Figure 3: Trait localization with other variants of HSPS and AQ questionnaires. a, Same format as Figure 3b but when the short version of AQ and 25 questions in HSPS (discarding two uncategorized questions in Table 1 column 2) were used. b, Same format at Figure 3b but when the short version of AQ and the short version of HSPS (Table 1 column 3) were used.

Sup Figure.4

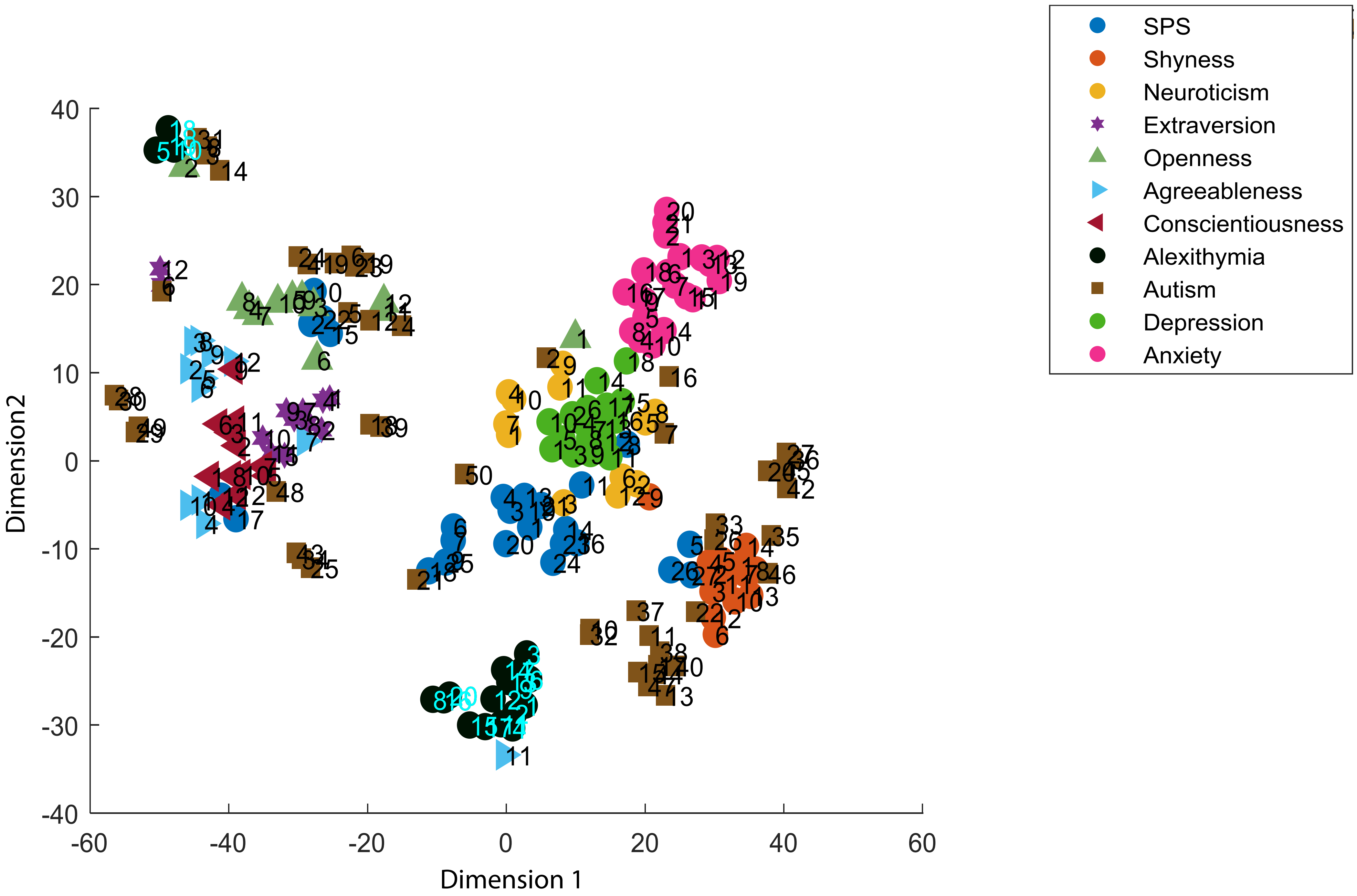

Supplementary Figure 4: Trait localization when using original autism score (0 or 1). Same format as Figure 3b but for the case when autism scores were 0 or 1 instead of 1, 2, 3, 4 used for all other analyses.

Sup Figure.5

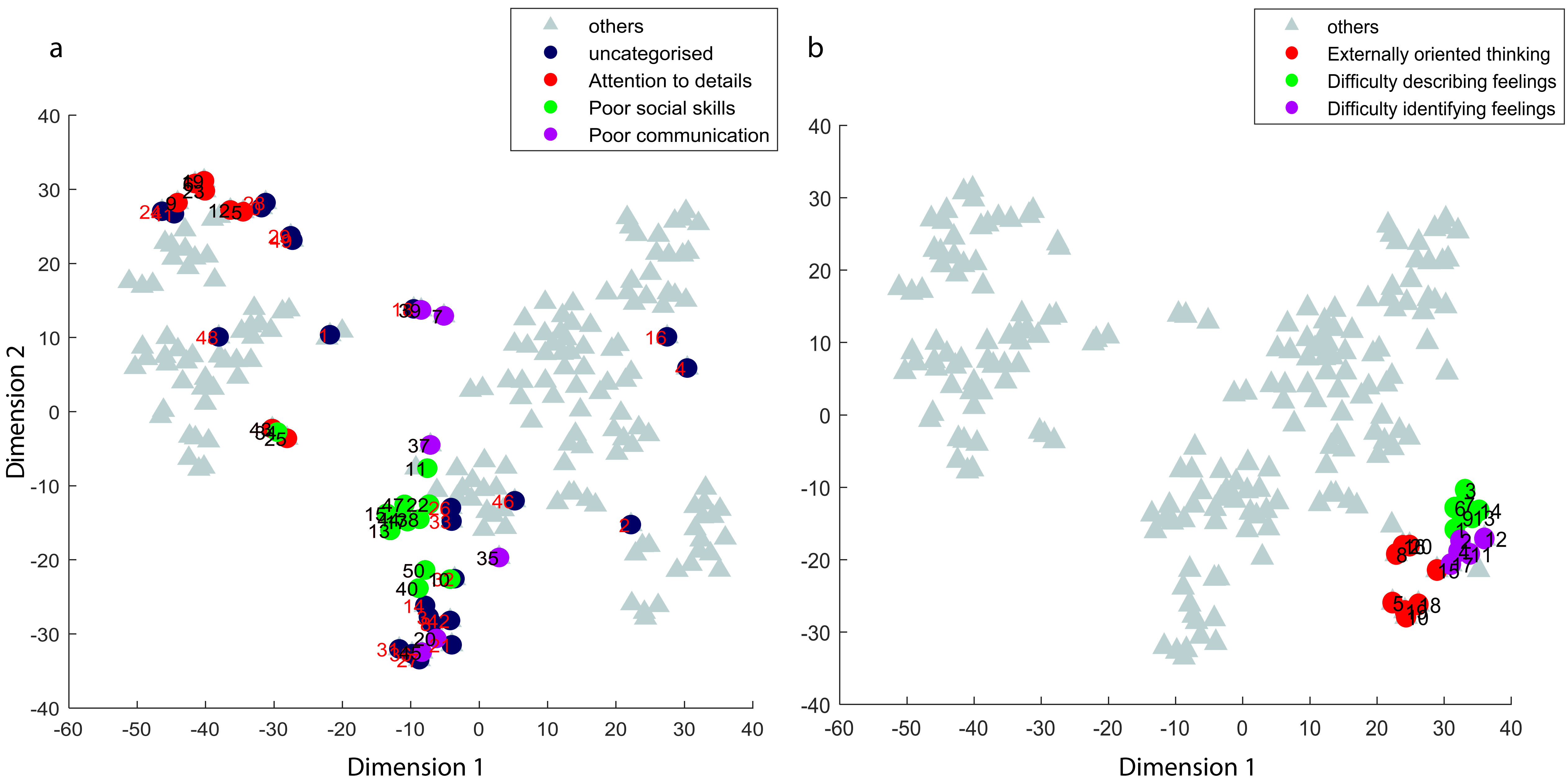

Supplementary Figure 5: Localization of autism and alexithymia subdomains in the trait space a, Same format as Figure 3c but for autism subdomains. b, Same format as Figure 3c but for alexithymia subdomains.

Sup Figure.6

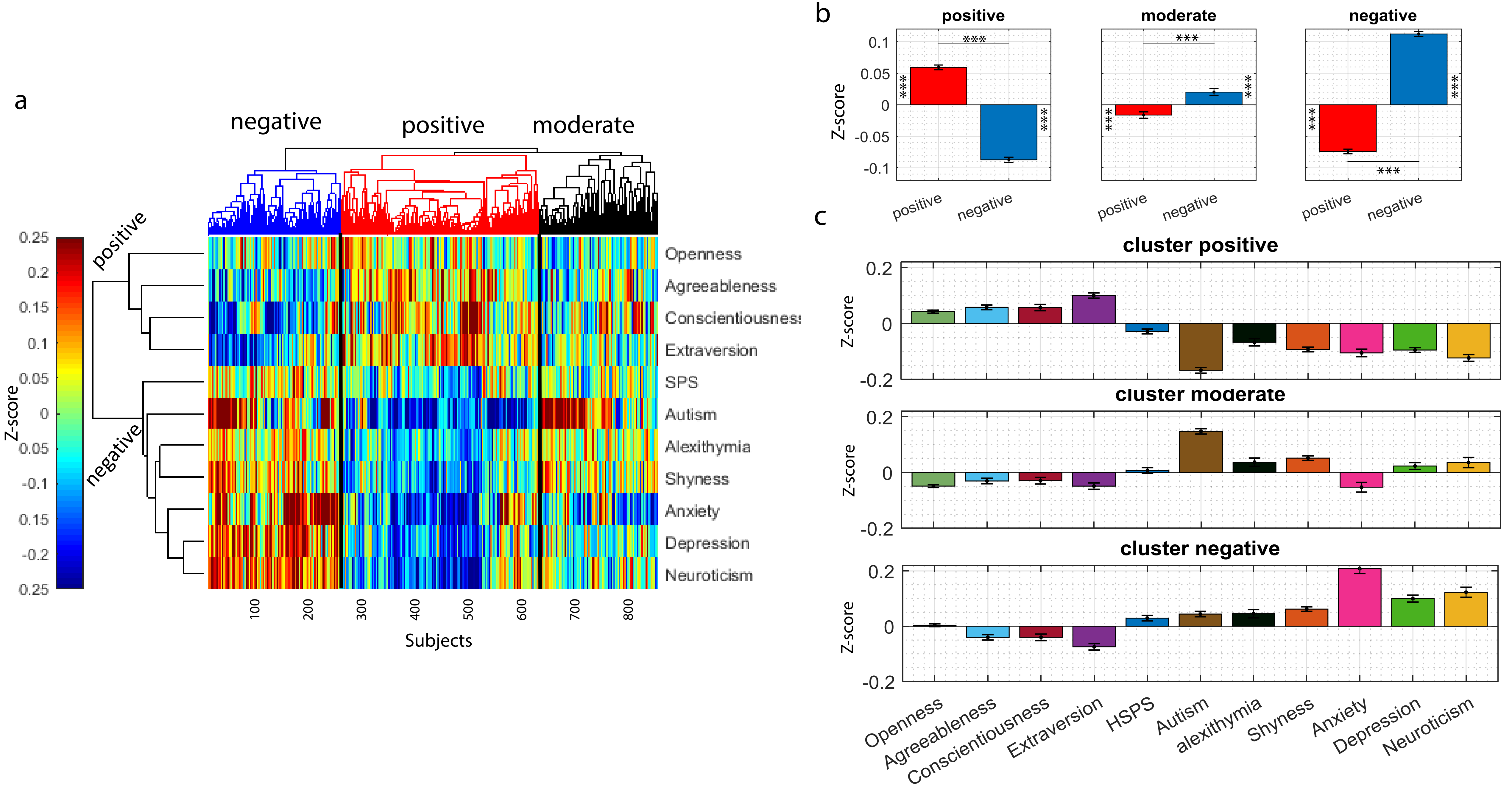

Supplementary Figure 6: Trait z-scores across all subjects and hierarchical clustering of traits and subjects for modified HSPS. a-c Same format as Figure1a-c but when z-scores for modified HSPS questionnaire (Table 1 column 4) was used. All other questionnaires are the same as Figure 1.

Sup Figure.7

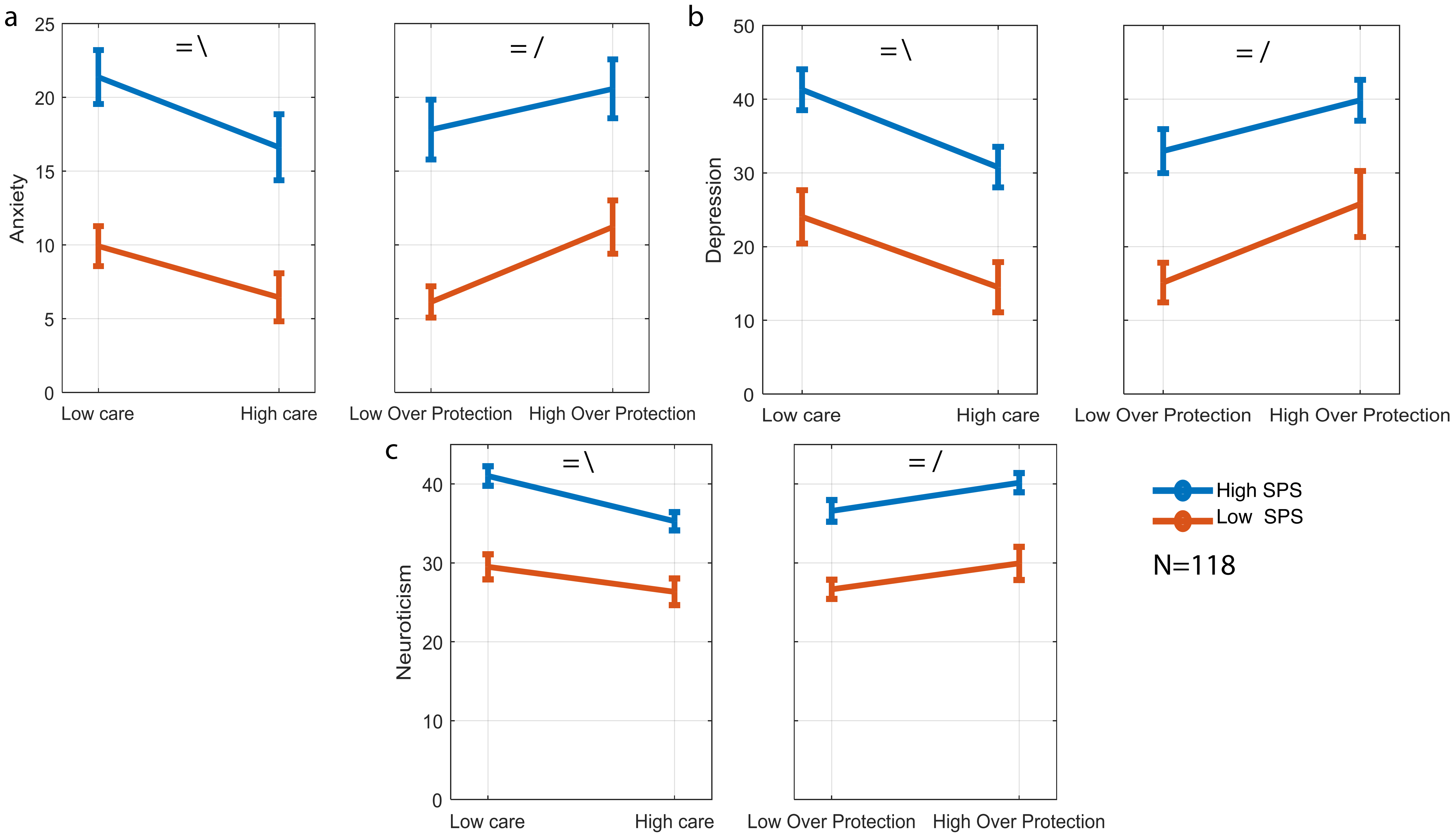

Supplementary Figure 7: Effect of SPS and PBI (care and overprotection) on anxiety, depression and neuroticism. a, Anxiety score: main effect of SPS $F_{1,114}>27.31$, $p<0.0$, main effect of care $F_{1,114}=4.2$, $p=0.04$, SPS x care interaction $F_{1,114}=0.09$, $p=0.76$, main effect of overprotection $F_{1,114}=3.76$, $p=0.05$, SPS x overprotection interaction $F_{1,114}=0.33$, $p=0.56$, b, Depression score: main effect of SPS $F_{1,114}>23.06$, $p<0.0$, main effect of care $F_{1,114}=10.06$, $p=0.001$, SPS x care interaction $F_{1,114}=0.0$, $p=0.95$, main effect of overprotection $F_{1,114}=7.74$, $p=0.006$, SPS x overprotection interaction $F_{1,114}=0.45$, $p=0.50$ c, Neuroticism score: main effect of SPS $F_{1,114}>47.59$, $p<0.0$, main effect of care $F_{1,114}=9.4$, $p=0.002$, SPS x care interaction $F_{1,114}=0.81$, $p=0.36$, main effect of overprotection $F_{1,114}=5.33$, $p=0.02$, SPS x overprotection interaction $F_{1,114}=0.02$, $p=0.90$. =, significant main effect of SPS, \ significant main effect care or overprotection, x significant interaction. Error bars are s.e.m. here and through-out.

Sup Figure.8

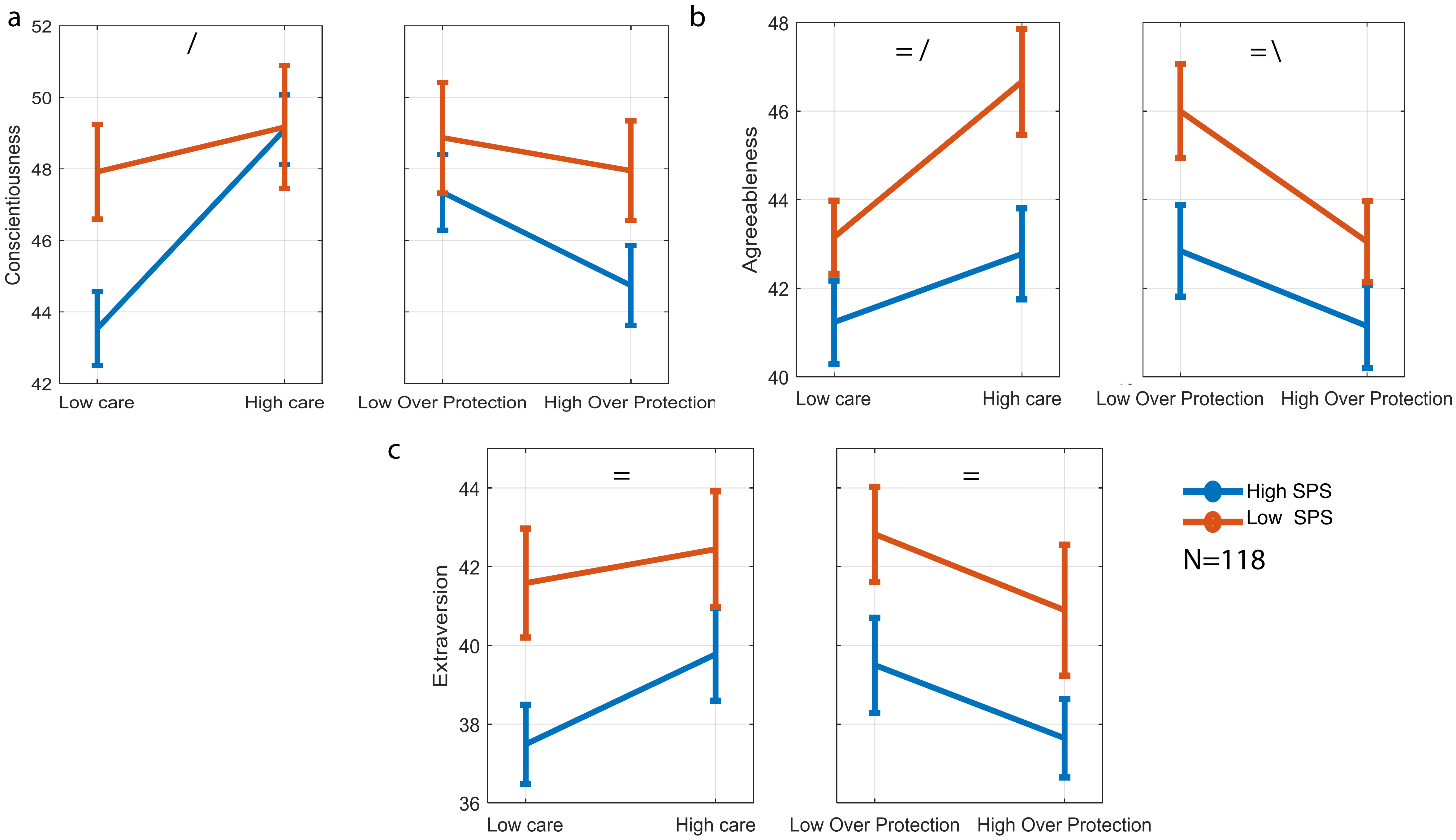

Supplementary Figure 8: Effect of SPS and PBI (care and overprotection) on conscientiousness, agreeableness and extraversion scores. a, conscientiousness score: main effect of SPS $F_{1,114}>3.08$, $p<0.08$, main effect of care $F_{1,114}=7.61$, $p=0.006$, SPS x care interaction $F_{1,114}=2.89$, $p=0.09$, main effect of overprotection $F_{1,114}=1.97$, $p=0.16$, SPS x overprotection interaction $F_{1,114}=0.38$, $p=0.53$, b, agree-ableness score: main effect of SPS $F_{1,114}>5.71$, $p<0.01$, main effect of care $F_{1,114}=5.61$, $p=0.01$, SPS x care interaction $F_{1,114}=0.85$, $p=0.35$, main effect of overprotection $F_{1,114}=4.83$, $p=0.03$, SPS x overprotection interaction $F_{1,114}=0.35$, $p=0.55$, c, extraversion score: main effect of SPS $F_{1,114}>6.97$, $p<0.01$, main effect of care $F_{1,114}=1.6$, $p=0.20$, SPS x care interaction $F_{1,114}=0.3$, $p=0.58$, main effect of overprotection $F_{1,114}=2.29$, $p=0.13$, SPS x overprotection interaction $F_{1,114}=0$, $p=0.97$, significant main effect of SPS, \ significant main effect care or overprotection, x significant interaction.

Sup Table.1

Participants' demographics.

N	837
Gender	
Females	68.57%
Males	31.43%
Mean age (SD)	28.12 (9.64)
Nationality	
Iranian	66.06%
Turkish	15.53%
Kurdish	6.33%
Afghan	2.51%
Arab	0.25%
Others	9.31%
Education	
High school	9.70%
High school Diploma	30.34%
Bachelor's degree	37.51%
Master's degree	19.95%
Physician	0.59%
Doctorate's degree	1.91%

Sup Table.2

Graph connectivity and centrality of traits. Number and sign of connections of each node with nodes in the positive cluster (2nd column) and with the negative cluster (3rd column) for the graph shown in Figure 2b. Rows are grouped into negative cluster traits (top 7 rows) and positive cluster traits (bottom 4 rows) and sorted by centrality within each trait as a node in the graph (Methods).

connection	with positive nodes	with Negative nodes	sum connection	centrality
Neuroticism	2 negative	4 positive	6	1.58
Shyness	1 negative	3 positive	4	1.17
Depression	1 negative	3 positive	4	1.16
Alexithymia	3 negative	2 positive	5	1.13
SPS	1 positive	3 positive	4	0.78
Autism	1 negative	2 positive	3	0.69
Anxiety	0	3 positive	3	0.58
Conscientiousness	3 positive	2 negative	5	0.80
Agreeableness	1 positive	3 negative	4	0.70
Extraversion	1 positive	2 negative	3	0.64
Openness	1 positive	1 positive, 1negative	3	0.62

Sup Table.3 Modified 16-item HSPS questionnaire.

our proposed 16-item SPS scale
1. Intense sensory stimuli make me overwhelmed
3.Other people's mood and behavior affects me
4. I am hypersensitive about pain
6. I am sensitive about the effects of caffeine in tea and coffee.
7.I'm easily influenced by stuff like bright light, pungent odors, and rough fabrics with rough texture, and the closed siren sound.
9. I get annoyed by loud noises
13. I get scared and panicked easily.
14. I feel stressed when I have a lot of work to do in a short period of time.
16.I get annoyed when others try to force me to do a lot of things
18. In my opinion, we should avoid watching violent movies.
19. I get irritated when a lot happens around me.
20. Hunger triggers a powerful reaction in me and perturbs my mood and concentration.
21. The changes in life make me feel stressed.
23. I find it unpleasant to do all the things once.
24. my priority is to avoid difficult and uncomfortable situations, to put my life in order.
25.Intense motives such as loud noises or deranged scenes disturb me.

Sup Table.4

Subject group membership confusion matrix for 27-item (original) and modified 16-Item HSPS questionnaire. Confusion matrix showing the number of subjects in positive, moderate and negative groups when using the original 27-item HSPS questionnaire (rows) and modified HSPS questionnaire (columns). Squares are color-coded proportional to the number shown in them for ease of visualization.

Original/Modified SPS	positive	moderate	negative	sum
positive	290	28	10	328
moderate	51	182	63	296
negative	28	11	174	213
sum	369	221	247	837

Sup Table.5 Pairwise correlation matrix of SPS, depression, anxiety, parental care and parental over protection.

	SPS	Depression	Anxiety	PBI care	PBI Over Protection
SPS	1	0.52**	048**	-0.052	0.18**
Depression		1	0.64**	-0.3**	0.28**
Anxiety			1	-0.24**	0.17**
PBI care				1	-0.59**
PBI Over Protection					1

**p<0.01
